# Supplementary material for: Identification and Biological Characteristics of Mortierella alpina Associated with Chinese Flowering Cherry (Cerasus serrulata) Leaf Blight in China
Source: J Fungi (Basel). 2024 Jan 5;10(1):50. doi: 10.3390/jof10010050 (PMC10817311; doi:10.3390/jof10010050)
Supplement: Supplementary file 1 [file jof-10-00050-s001.zip › jof-2784244-supplementary.pdf]

Table S1. A brief description of the 26 isolates obtained from leaves with typical symptoms of *C. serrulata*.

Strains	Source of isolation	Colony morphology on PDA Medium	Pathogenicity
XA-1	*Cerasus serrulata*	yellow, dense aerial mycelium, regular margin, flat colonies, smooth surface	-
XA-2	*Cerasus serrulata*	white, floccose and dense aerial mycelia, plain and flat at the regular edges	-
XA-3	*Cerasus serrulata*	white, floccose and dense aerial mycelia, plain and flat at the regular edges	-
XA-4	*Cerasus serrulata*	cottony white, fluffy aerial mycelium, mycelia sparsed, plain and flat at the regular edges	-
XA-5	*Cerasus serrulata*	yellow, dense aerial mycelium, regular margin, flat colonies, smooth surface	-
XA-6	*Cerasus serrulata*	white, floccose and dense aerial mycelia, plain and flat at the regular edges	-
XA-7	*Cerasus serrulata*	cottony white, fluffy aerial mycelium, mycelia sparsed, plain and flat at the regular edges	-
XA-8	*Cerasus serrulata*	yellow, dense aerial mycelium, regular margin, flat colonies, smooth surface	-
XA-9	*Cerasus serrulata*	cottony white, fluffy aerial mycelium, mycelia sparsed, plain and flat at the regular edges	-
XA-10	*Cerasus serrulata*	milky white, cottony aerial mycelium, wavy edge, raised on the surface	+
XA-11	*Cerasus serrulata*	white, floccose and dense aerial mycelia, plain and flat at the regular edges	-
XA-12	*Cerasus serrulata*	brown to cottony white at the edges, dense aerial mycelia, flat colonies, smooth surface, regular margin	-
XA-13	*Cerasus serrulata*	white, floccose and dense aerial mycelia, plain and flat at the regular edges	-
XA-14	*Cerasus serrulata*	brown to cottony white at the edges, dense aerial mycelia, flat colonies, smooth surface, regular margin	-
XA-15	*Cerasus serrulata*	milky white, cottony aerial mycelium, wavy edge, raised on the surface	+
XA-16	*Cerasus serrulata*	brown to cottony white at the edges, dense aerial mycelia, flat colonies, smooth surface, regular margin	-
XA-17	*Cerasus serrulata*	yellow, dense aerial mycelium, regular margin, flat colonies, smooth surface	-
XA-18	*Cerasus serrulata*	milky white, cottony aerial mycelium, wavy edge, raised on the surface	+
XA-19	*Cerasus serrulata*	cottony white, fluffy aerial mycelium, mycelia sparsed, plain and flat at the regular edges	-
XA-20	*Cerasus serrulata*	yellow, dense aerial mycelium, regular margin, flat colonies, smooth surface	-
XA-21	*Cerasus serrulata*	yellow, dense aerial mycelium, regular margin, flat colonies, smooth surface	-
XA-22	*Cerasus serrulata*	brown to cottony white at the edges, dense aerial mycelia, flat colonies, smooth surface, regular margin	-
XA-23	*Cerasus serrulata*	yellow, dense aerial mycelium, regular margin, flat colonies, smooth surface	-
XA-24	*Cerasus serrulata*	brown to cottony white at the edges, dense aerial mycelia, flat colonies, smooth surface, regular margin	-
XA-25	*Cerasus serrulata*	brown to cottony white at the edges, dense aerial mycelia, flat colonies, smooth surface, regular margin	-
XA-26	*Cerasus serrulata*	yellow, dense aerial mycelium, regular margin, flat colonies, smooth surface	-
